# Analgesic efficacy of meloxicam vs ibuprofen on pain after third molar surgery in adult patients. A randomized controlled clinical trial

**DOI:** 10.4317/medoral.27543

**Published:** 2025-10-14

**Authors:** Ruth Falcón-Perez, Vivian Pradenas-Loaiza, Carla Bertrán-Delgado, Felipe-Rodrigo Aguilera, Juan Kunstmann-Camino

**Affiliations:** 1 School of Dentistry, Faculty of Medicine, Universidad Austral de Chile, Valdivia, Chile

## Abstract

**Background:**

This randomized clinical trial was designed to evaluate the postoperative analgesic efficacy of ibuprofen compared to meloxicam in patients undergoing third molar extraction.

**Material and Methods:**

Sixty-eight patients who had been indicated for the extraction of both a maxillary (semi-erupted or fully erupted crown) and mandibular third molar (Class I, Position A, and Class I, Position B, according to Pell and Gregory's classification) were randomly assigned to receive either meloxicam (7.5 mg every 12 hours, n = 34) or ibuprofen (400 mg every 8 hours, n = 34) following a single surgical procedure. Postoperative pain intensity was assessed using a Visual Analog Scale (VAS). All outcome measures were recorded during the first seven consecutive postoperative hours, and subsequently at 24, 48, and 72 hours. Addition, the presence or absence of adverse effects was recorded.

**Results:**

In this clinical trial, a total of 68 patients (mean age 24.7 years) completed the study in this clinical trial, and no patients were lost to follow-up. The results showed lower pain intensity in the ibuprofen-treated group at all evaluated time points, except at 72 hours; however, statistically significant differences were observed only at the 2-hour mark (p &lt; 0.05). Both groups exhibited a sustained decrease in pain from 24 hours postoperatively onward (VAS &lt; 2). Only two cases of mild dizziness were reported in the ibuprofen group.

**Conclusions:**

These findings suggest that both therapeutic regimens are effective and well-tolerated options for postoperative pain management in third molar extraction.

## Introduction

Third molar surgery is a common clinical procedure that involves trauma to the periodontal tissues, resulting in postoperative pain that requires careful and effective management. To alleviate this pain, nonsteroidal anti-inflammatory drugs (NSAIDs) have been widely used, demonstrating their efficacy in controlling pain and inflammation associated with the surgical process (1).

NSAIDs have been shown to be effective in the treatment of mild to moderate postoperative pain (2). The mechanism of action of these drugs involves the reversible inhibition of the cyclooxygenase (COX) enzyme, which has two isoforms, COX-1 and COX-2, that mediate the production of prostaglandins and thromboxane A2. Prostaglandins regulate various physiological functions and play a key role in inflammatory and nociceptive processes (1 , 3).

Ibuprofen is a non-selective NSAID, which is often used as a control medication after oral surgery and that provides relatively equivalent inhibition of both COX-1 and COX-2 isoforms (4). This is one of the most frequently administered drugs following the surgical extraction of third molars and is often used as a positive control in clinical trials due to its analgesic efficacy and good tolerance (5). Additionally, it is nearly free of complications or adverse effects (6 , 7). Some studies have shown that the analgesic effect of ibuprofen does not increase with doses exceeding 400 mg (8 , 9).

On the other hand, meloxicam is preferentially selective for COX-2 but also inhibits COX-1 (10). Is primarily used for the treatment of acute and chronic painful, inflammatory, and degenerative disorders, including rheumatoid arthritis, osteoarthritis, ankylosing spondylitis, and juvenile idiopathic arthritis. It is administered as an oral dose of 7.5 mg, which can be increased if necessary, to a maximum of 15 mg per day (11 , 12).

Currently, in the field of dentistry, there is a need for evidence regarding the use of meloxicam for postoperative pain management in dental surgeries (13). Given the aforementioned, it is important to conduct clinical studies comparing the analgesic efficacy of these medications as an alternative for postoperative pain relief. The aim of this study, was to compare the postoperative analgesic efficacy of meloxicam (7.5 mg) versus ibuprofen (400 mg) in patients undergoing third molar extractions.

The null hypothesis was that there would be no difference between the two analgesic drugs tested.

## Material and Methods

A double-blind, randomized clinical trial with parallel groups was conducted following the recommendations of the CONSORT guidelines.

The participants in this study were patients treated at the Dental Clinic of Universidad Austral de Chile (Valdivia, Chile) who had been indicated for the extraction of a maxillary and mandibular third molar between April and July 2024.

This study was approved by the Scientific Ethics Committee of the Servicio de Salud Los Ríos (CEC-SSLR) and adhered to the ethical standards of the Declaration of Helsinki (World Medical Association, 2017). The study was registered at ClinicalTrials.gov (NCT06704113).

The study included patients who agreed to participate and met the following criteria:

Inclusion criteria

1. Adult participants aged 18 to 59 years with ASA Class I health status.

2. Non-smokers.

3. No history of pericoronitis or signs of inflammation in the last 30 days.

4. Maxillary third molars with a semi-erupted or fully erupted crown.

5. Mandibular third molars classified as Class I, Position A, and Class I, Position B, according to Pell and Gregory's classification (Pell et al., 1993).

6. Good oral hygiene.

Exclusion criteria

1. Subjects with maxillary and/or mandibular third molars with a fully impacted crown or periodontal involvement (gingivitis or periodontitis).

2. Pregnant or breastfeeding women

3. Patients who self-report hypersensitivity to NSAIDs.

4. Subjects who have taken any anti-inflammatory medication in the last 7 days.

5. Subjects with a history of drug abuse.

6. Surgical intervention lasting more than 45 minutes.

7. Any pre-existing pain or acute inflammatory or infectious conditions.

Participant enrollment was conducted by two researchers (R.F. and V.P.), who selected voluntary participants based on the aforementioned criteria.

The required sample size was determined according to the recommendations of Vallecillo et al. (14) using a 95% confidence interval, a statistical power of 90%, a standard deviation of 2.5, and a 1:1 ratio between groups. Based on these parameters, the sample size was set at 68 patients, with 34 in each group.

Randomization

Patients who met the selection criteria were randomly assigned to two groups. Group A (control) received a 400 mg ibuprofen capsule (Laboratorio Chile | Teva) every 8 hours for the first 72 hours after extraction, while Group B (experimental) received a 7.5 mg meloxicam capsule (Laboratorio Pasteur, Chile) every 12 hours for the same period.

Group allocation was performed using a balanced internet-based randomization system (Available at: www.randomization.com). The assignments were stored in consecutively numbered, opaque, sealed envelopes, which contained the pain registration questionnaire along with the medication, dispensed in pill organizers, and accompanied by usage instructions.

Blinding

The medication administered to each study participant was blinded to the patient, the surgeon, and the clinical investigator responsible for follow-up and outcome assessments.

Before each intervention, one of the authors, who was not involved in data recording or analysis, assigned the envelopes labeled with both treatment methods. These envelopes were stored in a box containing a paper indicating either "A" or "B." After the extraction, envelopes were selected for each patient, and one of the authors was responsible for distributing the treatment groups. A single operator, who was unaware of the previously recorded data, performed all procedures.

Surgical procedure

All surgical procedures were conducted by the same surgeons. Immediately before the intervention, patients rinsed their mouths for 2 minutes using 10 mL of 0.12% chlorhexidine mouthwash (Perio-Aid; Dentaid, Barcelona, Spain). All surgeries were performed on an outpatient basis under local anesthesia using 2% lidocaine with 1:100,000 epinephrine (Ultracain; Normon, Madrid, Spain) for inferior alveolar nerve block and posterior superior alveolar nerve block (with additional palatal anesthesia). The surgical procedures were standardized and primarily consisted of syndesmotomy, grasping, luxation, and avulsion, followed by alveolar exploration and irrigation with 10 mL (0.9%) saline solution. The procedure was completed with a simple suture technique (3/0 chromic catgut, TAGUM) and compression of the surgical wound with sterile gauze for 40 minutes. Patients not feeling adequate pain relief at 1 hour after taking the study medication received 1 g paracetamol as rescue analgesic.

All participants received post-extraction instructions both verbally and in writing. In the event of any postoperative complications or adverse effects, patients were instructed to notify the responsible researchers (R.F. and V.P.) via telephone. Urgent care was provided by a dentist at the Dental Clinic of the Austral University of Chile (Valdivia).

Outcome measures

Patient-related variables of gender and age were recorded. The primary outcome variable was postoperative pain intensity by visual analogue scale (VAS) on a 10 (0="no pain" and 10="worst possible pain") at 1, 2, 3, 4, 5, 6, 7, 24, 48 and 72 h postoperatively. Pain intensity was recorded by each patient using a questionnaire specifically designed for this study, which was provided after the extraction to be completed at home. To minimize recall bias in data recording and medication administration, the researchers were responsible for contacting participants via telephone at designated times to remind them to take the assigned medication and to document pain intensity. In addition, the presence or absence of adverse effects was recorded (nausea, vomiting, somnolence, dizziness, trembling, sweating, dyspepsia, diarrhea, bleeding, disorientation).

Statistical analysis

Descriptive statistics were used to present the data. Differences in patient-related variables were assessed using the chi-square test and the Kruskal-Wallis test. To evaluate postoperative pain intensity following third molar extraction between both treatment groups, a repeated-measures ANOVA was performed, followed by Tukey's post hoc test. The significance level was set at p&lt;0.05. All statistical analyses were conducted using RStudio (v. 1.3.959), and graphical representations were generated using Prism 9 (for macOS, v. 9.0.1).

## Results

**Figure 1:**
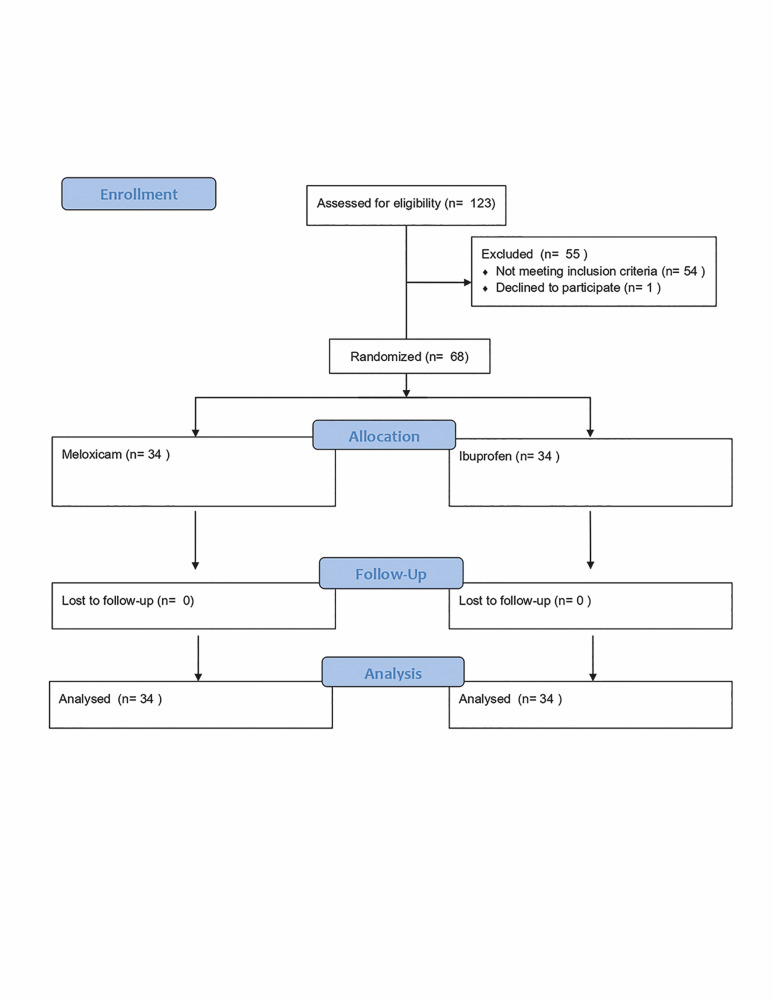
Flow diagram depicting the progression of subjects through the phases of a randomized study.

The distribution of patients based on the administered medication consisted of 34 patients in each group. Of the 68 patients randomly assigned to the two treatment groups, 46 (67.6%) were women and 22 (32.4%) were men. No patients were lost to follow-up. The flow of patients through the study is depicted in Figure 1.

1

The age range was between 18 and 45 years, with a mean age of 24.7±5.39 years. A total of 52 maxillary and 16 mandibular third molars were extracted in the meloxicam group, whereas 43 maxillary and 25 mandibular third molars were extracted in the ibuprofen group.

When analyzing the patient-associated variables, pain intensity according to gender, measured using the VAS, was lower in men from the ibuprofen-treated group (1.5±1.6; 95% CI 1.12-1.85). However, no significant differences were found between the treatment groups regarding gender (p=0.98). Additionally, age group analysis showed that most patients were within the 18-22 and 23-27 year ranges. The mean pain intensity was lower in the ibuprofen-treated group compared to the meloxicam group. Analysis using the Kruskal-Wallis test revealed statistically significant differences (p=0.00183). Details of the patient-associated variables are shown in Table 1.

Table 1

During the first hour, the mean postoperative pain perception reached the highest values, gradually decreasing in the following hours. These values were 2.74±2.59; 95% CI 1.83-3.64 for ibuprofen and 3.82±2.72; 95% CI 2.87-4.77 for meloxicam. Only during the second hour were significant differences observed (p&lt;0.05) between the ibuprofen and meloxicam groups (2.32 ± 2.23; 95% CI 1.54-3.10 vs 3.65±2.47; 95% CI 2.78-4.51, respectively). The details of the different time (hours) evaluated are shown in Table 2.

Table 2

**Figure 2:**
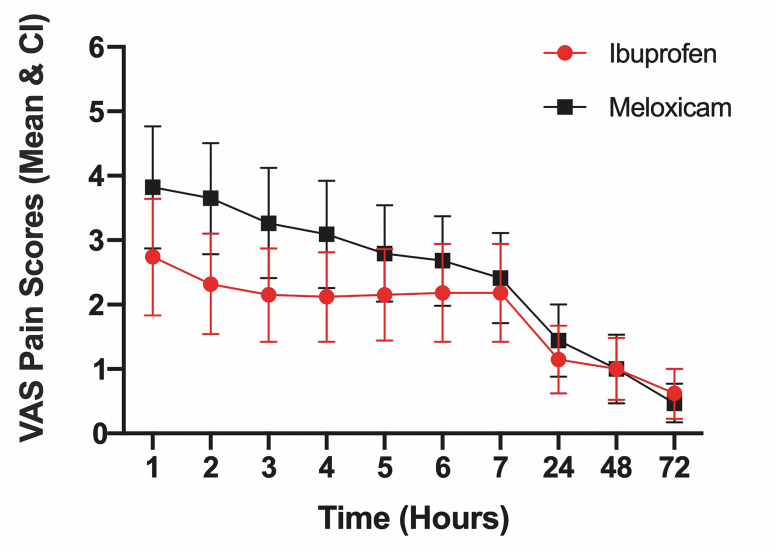
Postoperative VAS pain measures between ibuprofen and meloxicam groups.

VAS pain values were lower in the ibuprofen group at all time points except at 72 hours. From 24 hours to 72 hours post-surgery, a considerable decrease in postoperative pain intensity was observed, reaching the lowest mean values (VAS&lt;2) in both groups (Figure 2).

2

Only two cases of mild dizziness were reported in the ibuprofen group, and no patients required the use of rescue analgesics.

## Discussion

The surgical removal of third molars, similar to other invasive procedures, often elicits inflammatory responses and postoperative pain. This underscores the importance of designing well-structured clinical trials that compare the analgesic efficacy of various pharmacological agents for effective postoperative pain management.

NSAIDs have proven to be effective in the management of mild to moderate postoperative pain and are considered a first-line analgesic therapy for third molar surgery (15 , 16). Our results showed that following third molar surgery, the ibuprofen group (400mg) exhibited lower mean pain scores (VAS) during the first 7 hours compared to the meloxicam group (75mg); however, these differences were not statistically significant, except at the second hour (2.32±2.23 vs. 3.65±2.47, respectively). A notable reduction in postoperative pain intensity was observed from the 24-hour mark onward. Both ibuprofen and meloxicam proved to be effective in reducing postoperative pain in patients undergoing third molar extractions.

Pain intensity was measured using VAS, on a 0 to 10 scale, which is analogous in structure and validity to the traditional 0 to 100mm scale. According to Jensen et al. (17) a mean score below 40mm is considered indicative of mild pain. In our study, we observed a mean VAS score of less than 4 during the first hour for both the ibuprofen and meloxicam groups (2.74; 95% CI 1.83-3.64 and 3.82; 95% CI 2.87-4.77, respectively), with pain intensity progressively decreasing up to 72 hours postoperatively.

Ibuprofen 400mg in oral administration is commonly chosen in many clinical trials to compare other analgesics' efficacy in pain relief following third molars surgery (3 , 18) It has been shown to effectively manage postoperative pain in adults due to rapid absorbability, peak plasma concentration in 1-2 hours, and long-lasting effects up to 6 hours (19).

A Cochrane review assessed the effectiveness of ibuprofen versus paracetamol for pain relief following the surgical extraction of impacted lower third molars. Most of the included studies compared 400 mg of ibuprofen with 1000 mg of paracetamol, which are the dosages most commonly prescribed in clinical practice. The findings indicated that ibuprofen was more effective in managing postoperative pain. Moreover, the combination of both drugs demonstrated promising outcomes, showing superior pain control compared to either medication alone, as reported in two clinical trials (18).

Other studies have evaluated the use of ibuprofen for pain control. Vallecillo et al. (14) compared the administration of 75mg of tramadol hydrochloride plus 25mg of dexketoprofen versus 400mg of ibuprofen as monotherapy. Pain intensity was monitored over a 48-hour follow-up period. Although pain scores measured using the VAS were consistently lower in the tramadol-dexketoprofen group, the differences were not statistically significant at any of the assessment time points. Akinbade et al. (20) compared the effects of ibuprofen, celecoxib and tramadol on pain after surgical extraction of impacted mandibular third molars. The mean VAS score of the celecoxib group (32.35±SD 23.96) at 4 hours was the lowest among the three groups. This was followed by the ibuprofen group with mean VAS score of 38.96±SD 22.30. Whereas, the subjects in tramadol group experienced the highest VAS score (53.31±SD 23.30) at 4 hours. There was statistically significant difference in the mean VAS scores at 4 hours after extraction when the three groups were compared (p=0.0039). Celecoxib group also had the lowest mean VAS scores at 8 hours, 24 hours and 48 hours after the extraction.

Meloxicam has been considered a suitable pharmacological alternative for oral surgical procedures due to its analgesic and anti-inflammatory effects, along with a reduced incidence of side effects. Is a preferential COX-2 inhibitor, which results in a lower incidence of gastrointestinal side effects compared to non-selective NSAIDs (21 , 22). It is almost completely, though slowly, absorbed, with a tmax of approximately 5-6 hours. Its long elimination half-life, around 20 hours, allows for once-daily dosing, which enhances patient adherence due to its convenient administration schedule (23 , 24).

Oral meloxicam (15mg) has been compared with other NSAIDs such as diclofenac (100mg). The results of this study showed that patients who received 15mg of meloxicam experienced significantly less postoperative pain (p=0.04) and a greater reduction in trismus compared to those who received 100mg of diclofenac (p=0.03). The meloxicam group also exhibited less postoperative swelling than the diclofenac group; however, this difference was not statistically significant (25). A study evaluated the efficacy of administering meloxicam at doses of 7.5mg or 15mg once daily for four days following third molar extraction. The results indicated that pain, trismus, and inflammation associated with the extraction of lower third molars without the need for osteotomy can be effectively managed with 7.5mg of meloxicam once daily. Nevertheless, for more aggressive extractions, a 15 mg dose is recommended to achieve more adequate symptomatic control (13).

Additionally, Christensen et al. (26) evaluated the efficacy, safety, and tolerability of intravenous (IV) meloxicam in the control of postoperative pain following surgical extraction of more than two third molars, at least one of which involved partial or complete mandibular bony removal. Single doses of IV meloxicam (15mg, 30mg, and 60mg) resulted in significant pain reduction compared to placebo, with the 60mg dose being the most effective. Moreover, the 30mg and 60mg IV meloxicam doses demonstrated statistically significant superiority over oral ibuprofen (400mg) in terms of pain reduction during the first 24 hours. Patients treated with IV meloxicam showed a lower need for rescue medication and reported greater overall satisfaction with the treatment. The absence of serious adverse events or treatment discontinuations due to side effects supports its favorable safety profile, positioning IV meloxicam as an effective and well-tolerated option for the management of acute postoperative pain.

The findings of our study indicate that both drugs are effective in managing postoperative pain following third molar surgery that does not involve osteotomy. In both groups, a progressive reduction in pain intensity was observed during the first 7 hours following the procedure, with a marked decrease between 24 and 72 hours after the surgical intervention. Once-daily administration of meloxicam may enhance patient adherence to postoperative medication by simplifying the dosing regimen.

Among the limitations of our study, it is important to note that most clinical trials assessing postoperative pain commonly involve impacted mandibular third molars, the use of preemptive analgesia, and single-dose administration. In contrast, the present study evaluated postoperative pain control in surgeries involving erupted third molars. The sample size was relatively small, although sufficient to detect statistically significant differences in postoperative pain scores between the two groups evaluated. An additional limitation was the absence of a placebo group in the trial, which means that a placebo effect in the reduction of pain intensity cannot be ruled out. Finally, pain assessment continues to represent a constant challenge due to its inherent subjectivity in patient perception.

## Conclusions

Considering the limitations of our study, both ibuprofen 400 mg and meloxicam 7.5 mg proved to have analgesic effects on postoperative pain after third molar surgery. However, in the ibuprofen group, a lower pain intensity was observed two hours postoperatively, suggesting a faster onset of action compared to meloxicam. Despite these differences, both drugs proved effective for postoperative pain management

## Figures and Tables

**Table 1:** Table Demographic profile of patients: age and gender.

Variable		Ibuprofen					Meloxicam					P-value
		N	%	Mean	SD	95% CI	N	%	Mean	SD	95% CI	
Sex	Male	12	35.3	1.5	1.6	[1.12-1.85]	10	29.4	2.3	1.2	[1.86-2.64]	0.98a
	Female	22	64.7	2.1	1.6	[1.79-2.34]	24	70.6	2.6	1.6	[2.24-2.86]	
Age group	18-22	12	35.3	2.8	1.9	[2.35-3.23]	12	35.3	3.3	1.3	[2.84-3.73]	0.00183b
	23-27	13	38.2	1.3	1.1	[0.98-1.54]	17	50	2.2	1.4	[1.83-2.48]	
	28-32	7	20.6	1.5	1.5	[1.12-1.91]	2	5.9	3.4	0.6	[2.59-4.21]	
	>32	2	5.9	1.4	1.8	[0.68-2.02]	3	8.8	0.3	0.1	[0.06-0.54]	
a Chi-Square test.												
b Kruskal-Wallis test.												

1

**Table 2:** Table Postoperative pain intensity (VAS) at the different times evaluated.

Pain intensity (VAS)	Ibuprofen		Meloxicam		P-value
	Mean±SD	95% CI	Mean±SD	95% CI	
At 1 h	2.74±2.60	[1.83-3.64]	3.82±2.72	[2.87-4.77]	0.1145
At 2 h	2.32±2.24	[1.54-3.10]	3.65±2.47	[2.78-4.51]	0.02123*
At 3 h	2.15±2.08	[1.42-2.87]	3.26±2.44	[2.41-4.12]	0.05797
At 4 h	2.12±2.00	[1.42-2.81]	3.09±2.38	[2.26-3.92]	0.08576
At 5 h	2.15±2.03	[1.44-2.86]	2.79±2.14	[2.05-3.54]	0.2022
At 6 h	2.18±2.18	[1.42-2.94]	2.68±2.00	[1.98-3.37]	0.2044
At 7 h	2.18±2.18	[1.42-2.94]	2.41±2.00	[1.71-3.11]	0.4931
At 24 h	1.15±1.50	[0.62-1.67]	1.44±1.60	[0.88-2.00]	0.3855
At 48 h	1.00±1.37	[0.52-1.48]	1.00±1.52	[0.47-1.53]	0.8635
At 72 h	0.62±1.10	[0.23-1.00]	0.47±0.86	[0.17-0.77]	0.6311

2

## Data Availability

The study was conducted by the Declaration of Helsinki and approved by the Research Ethics Committee of Servicio de Salud Los Ríos (CEC-SSLR, ORD Nº 95/2024).

## References

[1] Cetira Filho EL, Carvalho FSR, de Barros Silva PG, Barbosa DAF, Alves Pereira KM, Ribeiro TR (2020). Preemptive use of oral nonsteroidal anti-inflammatory drugs for the relief of inflammatory events after surgical removal of lower third molars: A systematic review with meta-analysis of placebo-controlled randomized clinical trials. J Craniomaxillofac Surg.

[2] Doleman B, Leonardi-Bee J, Heinink TP, Boyd-Carson H, Carrick L, Mandalia R (2021). Pre-emptive and preventive NSAIDs for postopera-tive pain in adults undergoing all types of surgery. Cochrane Database Syst Rev.

[3] Moore RA, Derry S, Aldington D, Wiffen PJ (2015). Single dose oral analgesics for acute postoperative pain in adults - an overview of Cochrane reviews. Cochrane Database Syst Rev.

[4] Orlando BJ, Lucido MJ, Malkowski MG (2015). The structure of ibuprofen bound to cyclooxygenase-2. J Struct Biol.

[5] Lustenberger FD, Grätz KW, Mutzbauer TS (2011). Efficacy of ibuprofen versus lornoxicam after third molar surgery: a randomized, double-blind, crossover pilot study. Oral Maxillofac Surg.

[6] Kellstein DE, Waksman JA, Furey SA, Binstok G, Cooper SA (1999). The safety profile of nonprescription ibuprofen in multiple-dose use: a meta-analysis. J Clin Pharmacol.

[7] Planas ME, Gay-Escoda C, Bagán JV, Santamaría J, Peñarrocha M, Donado M (1998). Oral metamizol (1 g and 2 g) versus ibuprofen and placebo in the treatment of lower third molar surgery pain: randomised double-blind multi-centre study. Cooperative Study Group. Eur J Clin Pharmacol.

[8] Jones K, Seymour RA, Hawkesford JE (1997). Are the pharmacokinetics of ibuprofen important determinants for the drug's efficacy in postoperative pain after third molar surgery?. Br J Oral Maxillofac Surg.

[9] Seymour RA, Ward-Booth P, Kelly PJ (1996). Evaluation of different doses of soluble ibuprofen and ibuprofen tablets in postoperative dental pain. Br J Oral Maxillofac Surg.

[10] Haffar A, Fillingham YA, Breckenridge L, Gursay D, Lonner JH (2022). Meloxicam versus Celecoxib for postoperative analgesia after total knee arthroplasty: Safety, Efficacy and Cost. J Am Acad Orthop Surg Glob Res Rev.

[11] Reginster JY, Distel M, Bluhmki E (1996). A double-blind, three-week study to compare the efficacy and safety of meloxicam 7.5 mg and meloxicam 15 mg in patients with rheumatoid arthritis. Br J Rheumatol.

[12] Wojtulewski JA, Schattenkirchner M, Barceló P, Le Loët X, Bevis PJ, Bluhmki E, et al. (1996). A six-month double-blind trial to compare the efficacy and safety of meloxicam 7.5 mg daily and naproxen 750 mg daily in patients with rheumatoid arthritis. Br J Rheumatol.

[13] Calvo AM, Sakai VT, Giglio FP, Modena KC, Colombini BL, Benetello V (2007). Analgesic and anti-inflammatory dose-response relationship of 7.5 and 15 mg meloxicam after lower third molar removal: a double-blind, random-ized, crossover study. Int J Oral Maxillofac Surg.

[14] Vallecillo C, Vallecillo-Rivas M, Gálvez R, Vallecillo-Capilla M, Olmedo-Gaya MV (2021). Analgesic efficacy of tramadol/dexketoprofen vs ibuprofen after impacted lower third molar extraction: a randomized controlled clinical trial. J Evid Based Dent Pract.

[15] Hersh EV, Moore PA, Grosser T, Polomano RC, Farrar JT, Saraghi M (2020). Nonsteroidal anti-Inflammatory drugs and opioids in postsurgical dental pain. J Dent Res.

[16] Barden J, Edwards JE, McQuay HJ, Wiffen PJ, Moore RA (2004). Relative efficacy of oral analgesics after third molar extraction. Br Dent J.

[17] Jensen MP, Martin SA, Cheung R (2005). The meaning of pain relief in a clinical trial. J Pain.

[18] Bailey E, Worthington HV, Van Wijk A, Yates JM, Coulthard P, Afzal Z (2013). Ibuprofen and/or paracetamol (acetaminophen) for pain relief after surgical removal of lower wisdom teeth. Cochrane Database Syst Rev.

[19] Derry CJ, Derry S, Moore RA, McQuay HJ (2009). Single dose oral ibuprofen for acute postoperative pain in adults. Cochrane Database Syst Rev.

[20] Akinbade AO, Ndukwe KC, Owotade FJ (2018). Comparative Analgesic Effects of Ibuprofen, Celecoxib and Tramadol after third Molar Surgery: A Randomized Double Blind Controlled Trial. J Contemp Dent Pract.

[21] Hawkey CJ (2001). COX-1 and COX-2 inhibitors. Best Pract Res Clin Gastroenterol.

[22] Ziesenitz VC, Welzel T, Van Dyk M, Saur P, Gorenflo M, Van Den Anker JN (2022). Efficacy and Safety of NSAIDs in Infants: A Comprehensive Review of the Literature of the Past 20 Years. Pediatr Drugs.

[23] Hawkey CJ (1999). COX-2 inhibitors. Lancet.

[24] Engelhardt G (1996). Pharmacology of meloxicam, a new non-steroidal anti-inflammatory drug with an improved safety profile through preferential inhibition of COX-2. Br J Rheumatol.

[25] Orozco-Solís M, García-Ávalos Y, Pichardo-Ramírez C, Tobías-Azúa F, Zapata-Morales JR, Aragon-Martínez OH (2016). Single dose of diclofenac or meloxicam for control of pain, facial swelling, and trismus in oral surgery. Med Oral Patol Oral Cir Bucal.

[26] Christensen SE, Cooper SA, Mack RJ, McCallum SW, Du W, Freyer A (2018). A Randomized Double-Blind Controlled Trial of Intravenous Meloxicam in the Treatment of Pain Following Dental Impaction Surgery. J Clin Pharmacol.

